# Addition of mental health to the lady health worker curriculum in Pakistan: now or never

**DOI:** 10.1186/s12960-023-00814-8

**Published:** 2023-04-18

**Authors:** Fauziah Rabbani, Samina Akhtar, Javeria Nafis, Shahid Khan, Sameen Siddiqi, Zul Merali

**Affiliations:** 1 Brain and Mind Institute, Aga Khan University, Karachi, Pakistan; 2 Department of Community Health Sciences, Aga Khan University, Stadium Road, P.O. Box 3500, Karachi, Pakistan; 3 Population and Public Health Stream, Aga Khan University, Stadium Road, Karachi, Pakistan

**Keywords:** Mental health, Lady health worker, mhGAP, Task shifting

## Abstract

The technical advisory group of the World Health Organization (Geneva, Switzerland) has suggested person-centered and community-based mental health services in response to the long-term and far-reaching mental health impacts of the COVID-19 pandemic. Task shifting is a pragmatic approach to tackle the mental health treatment gap in low- and middle-income countries. Pakistan is dismally resourced to address the mental health challenges. Pakistan’s government has established a lady health worker’s program (LHW-P) which can be effectively utilized to provide some basic mental health services at community doorsteps. However, lady health workers’ current curriculum does not include mental health as a subject. WHO’s Mental Health Gap Intervention Guide (mhGAP-IG) Version 2.0 for mental, neurological, and substance use disorders in non-specialist health settings can be adapted and utilized to be included as part of the LHW-P curriculum in Pakistan. Thus, the historical lack of access to mental health support workers, counsellors, and specialists can be addressed. Additionally, this will also help to reduce the stigma associated with seeking mental health care outside the boundaries of home, mostly at a huge cost.

## Background

Mental health impacts of the pandemic have been declared to be long-term and far-reaching. WHO’s technical advisory group suggested critical actions to be taken by national authorities in response to COVID-19. Of the many recommendations, one is to provide person-centered, community-based mental health services using innovative approaches [1]. Pakistan’s current estimated population is 210 million making it the 6th most populous country in the world with a growth rate of 2% per year. According to a report, there were 13,337 suicides in Pakistan in 2012 with a rate of 7.5 per 100,000 and a higher prevalence in women. The incidence and prevalence of suicide have increased by 2.6% when compared to the previous survey conducted in 2000 which is a major source of concern [2]. A systematic review concluded that 34% of Pakistanis had depression and anxiety disorders [3]. Reports further showed a 15% prevalence of adolescent and child mental health disorders, and around 4 million people using illicit drugs of which 70% were men within the age range of 15–40 [2].

Despite the high rate of psychiatric disorders in the country, help-seeking is very low due to prevailing stigma, lack of awareness, unemployment, poverty, and a low literacy rate [4]. Currently, only 0.4% of the national health budget is allocated to mental health. There are around 400 qualified psychiatrists in Pakistan, mostly concentrated in urban cities [5]. This creates a geographical disparity as 64% of Pakistan’s population resides in rural areas [6]. In addition, the country has only 5 major psychiatric hospitals, 650 inpatient mental health units, and 3800 outpatient clinics [7]. Studies have shown that due to limited awareness most people contact general practitioners when concerned about their mental health [2]. Moreover, people also resort to alternate sources such as spiritual healers in the absence of specialized mental health services.

WHO has advised low- and middle-income countries (LMICs) to tackle the mental health treatment gap through ‘task-shifting’, i.e., training non-specialists such as nurses, teachers, and community health workers (CHWs) to provide mental health services under the guidance of specialists [8]. This approach is pragmatic and sustainable but requires rapid deployment of public health initiatives, training programs, tech-enabled support, and data-gathering systems. A scoping review [9] supports the acceptability and effectiveness of adapting brief psychosocial treatments by non-specialist health workers in primary care and community-based settings for the management of common mental disorders in LMICs (Africa).

Santiago, Chile, has used stepped care approach successfully. This approach was a 3-month, multi-component intervention led by a group of non-medical health workers. They provided psychoeducation, structured and systematic follow-up, and medicines to 240 adult female primary care patients with severe depression. They found nearly 70% of patients recovered using a stepped care program at an additional cost of just 216 Chilean pesos (US$0.32) [10]. In a rural community in Pakistan with little access to mental health care, integration of cognitive behavior therapy (CBT) based intervention into the routine work of Lady Health Workers (LHWs) decreased the rate of depression in prenatally depressed women. LHWs received a short training on CBT and their feedback showed that it can be easily integrated in their routine work as almost all of the LHWs thought it was relevant to their day-to-day work and none of them considered it an extra burden [11]. A systematic review including 21 studies explored the views and experiences of service users and healthcare providers concluded that task-sharing mental health services in LMICs (Africa and South Asia) was largely considered acceptable and feasible [12]. Some mental health professionals, perhaps warned that therapists without qualifications in psychiatry or clinical psychology may compromise clinical standards and provide poor-quality care. However, this has been disproved by ample evidence demonstrating that the adoption of task-sharing models relying on non-specialist healthcare workers with proper training and supervision is capable of providing an efficacious and cost-effective approach to improving and scaling up mental health services [13].

Government of Pakistan established a CHWs’ program known as the Lady Health Worker-Programme (LHW-P) comprising LHWs and their Supervisors (LHSs) to strengthen health systems at the household and community levels and to connect local communities with hospital-based services. LHWs in Pakistan have minimum 8 years of education and undergo 15 months of classroom and field-based training. Each LHW has a designated catchment area of 100–150 households, catering to 1000 community members [14, 15]. A lady health supervisor (LHS) oversees the work of 20–25 LHWs on a routine basis, with her office typically located within a basic health unit (BHU) or a rural health center, which are the first-level healthcare facilities in Pakistan. A district program implementation unit coordinator oversees the work of all LHSs and LHWs in a district.

One of the key strengths of LHW-P is that LHWs are recruited from within local communities, and thus can deliver services in a culturally appropriate and acceptable manner. They have established acceptability in the community and are respected as a healthcare workforce. This workforce is responsible for providing basic preventive maternal, newborn, and child health (MNCH) and family planning services at community doorsteps and cover almost 60% of the rural population [14]. In recent years, apart from their main charter of MNCH services, LHWs and LHSs are participating in large health campaigns, community management of TB, Polio, and health education on HIV/AIDS [16].

### Mental Health Gap Intervention Guide: possible mental health curriculum for CHWs

For successful integration of mental health into primary healthcare in low and middle-income countries like Pakistan, grass-root level workers like LHWs need to acquire relevant knowledge and skills to recognize, refer and support people experiencing mental health disorders. They need to be trained in screening symptoms of mental ill health, communication skills, stress, and emotion management strategies. In this regard, WHO’s Mental Health Gap Intervention Guide (mhGAP-IG) version 2.0 for mental, neurological, and substance use disorders in non-specialist health settings [17] can be utilized and adapted to be included as part of the LHW-P curriculum.

Current curriculum of LHW-P includes health education, promotion of healthy behaviors, antenatal and postnatal care, and dispensing of ORS and Zinc [18]. Thus, the curriculum has the potential to include much needed aspects on promotion of mental health. This will address the historical lack of access to mental health support workers, counsellors, specialists, and reduce stigma associated with seeking mental health support and care mostly at a huge social and/or economic cost.

WHO recommends that mhGAP-IG should be adapted by countries to suit their local context, resources, and priorities [17]. This mhGAP-IG consists of ten priority conditions and besides a specialized health workforce is applicable and appropriate for training health technicians, community health workers and, in rare instances, traditional healers as well. There is growing evidence that mhGAP-IG can be used to successfully train non-specialized health workers in resource-limited settings to recognize and manage common mental illnesses [8]. In rural Rwanda, primary care nurses and CHWs were trained according to mhGAP guide for providing mental health services at primary care centers. The training was effective as a decline in psychological distress and functional impairment was observed [19]. In Pakistan, mhGAP curriculum was adapted to train primary healthcare physicians and psychosocial workers to provide mental health services in conflict-affected regions of North Waziristan [20].

Early results (yet to be published) of adapting mhGAP guide to the context of non-specialist LHWs in one district of Sind, Pakistan, showed that these front-line workers easily understood the causes, signs, and symptoms of common mental illnesses and found this guide useful in improving their communication and counselling skills for improving mental health [21]. CHWs can identify people with mental illnesses, provide psychosocial counselling at the community level and refer cases to the next level of care. Doctors working at BHU can administer pharmacological treatment in lieu of a psychiatrist. This approach allows to bridge the treatment gap in mental health with some quality control. Quality of service delivery for mental health care will, however, require frequent training and utilizing the existing LHWs’ supervisory mechanisms.

## Conclusion

Thus, to bridge the mental health delivery gap in Pakistan, a task-shifting approach is recommended. mhGAP guidelines offer a promising solution to build the mental health capacity of LHW-P. Once the content has been contextually and linguistically adapted considering the current mental health literacy of the LHWs, it has the potential to be a supplementary chapter in the LHW-P curriculum. A pilot feasibility study can later test the effectiveness of introducing mental health into LHW-P mandate and its impact on reducing mental distress.

Task-sharing models require increased numbers of human resources, adequate training, compensation for health workers who take on new mental health tasks, and ongoing structured supportive supervision at the community and primary health care level. It is now for the Government of Pakistan, academic institutions, non-governmental organizations, and development agencies to invest in implementing this approach to reduce the huge gap that is responsible for a substantial burden of disease in the country.

## Data Availability

Not applicable.

## Abbreviations

WHO: World Health Organization
COVID-19: Coronavirus disease of 2019
LMICs: Low and middle income countries
CHWs: Community health workers
LHW-P: Lady Health Worker-Programme
LHS: Lady health supervisor
LHW: Lady health worker
MNCH: Maternal, newborn, and child health
mhGAP-IG: Mental Health Gap Intervention Guide
